# Integration of a Geriatric Assessment With Intervention in the Care of Older Adults With Hematologic Malignancies

**DOI:** 10.3389/fonc.2021.775050

**Published:** 2021-12-08

**Authors:** Sarah A. Wall, Ying Huang, Ashleigh Keiter, Allesia Funderburg, Colin Kloock, Nicholas Yuhasz, Tanya R. Gure, Edmund Folefac, Erin Stevens, Carolyn J. Presley, Nicole O. Williams, Jessica L. Krok-Schoen, Michelle J. Naughton, Ashley E. Rosko

**Affiliations:** ^1^ Division of Hematology, The Ohio State University, Columbus, OH, United States; ^2^ The James Comprehensive Cancer Center, Columbus, OH, United States; ^3^ College of Medicine, The Ohio State University, Columbus, OH, United States; ^4^ Division of Geriatric Medicine, The Ohio State University, Columbus, OH, United States; ^5^ Division of Medical Oncology, The Ohio State University, Columbus, OH, United States; ^6^ Division of Palliative Medicine, The Ohio State University, Columbus, OH, United States; ^7^ College of Health Sciences, The Ohio State University, Columbus, OH, United States; ^8^ Division of Cancer Control and Prevention, The Ohio State University, Columbus, OH, United States

**Keywords:** geriatric assessment, oncogeriatrics, geriatric oncology, frailty, hematologic malignancy

## Abstract

The incidence of hematologic malignancies (HMs) is highest in the seventh decade of life and coincides with increasing occult, age-related vulnerabilities. Identification of frailty is useful in prognostication and treatment decision-making for older adults with HMs. This real-world analysis describes 311 older adults with HMs evaluated in a multidisciplinary oncogeriatric clinic. The accumulation of geriatric conditions [1-unit increase, hazards ratio (HR) = 1.13, 95% CI 1.00–1.27, p = 0.04] and frailty assessed by the Rockwood Clinical Frailty Scale (CFS, mild/moderate/severe frailty vs. very fit/well, HR = 2.59, 95% CI 1.41–4.78, p = 0.002) were predictive of worse overall survival. In multivariate analysis, HM type [acute leukemia, HR = 3.84, 95% CI 1.60–9.22, p = 0.003; myelodysplastic syndrome (MDS)/myeloproliferative neoplasm (MPN)/bone marrow failure, HR = 2.65, 95% CI 1.10–6.35, p = 0.03], age (per 5-year increase, HR = 1.46, 95% CI 1.21–1.76, p < 0.001), hemoglobin (per 1 g/dl decrease, HR = 1.21, 95% CI 1.05–1.40, p = 0.009), deficit in activities of daily living (HR = 2.20, 95% CI 1.11–4.34, p = 0.02), and Mini Nutrition Assessment score (at-risk of malnutrition vs. normal, HR = 2.00, 95% CI 1.07–3.73, p = 0.03) were independently associated with risk of death. The most commonly prescribed geriatric interventions were in the domains of audiology (56%) and pharmacy (54%). The Rockwood CFS correlated with prescribed interventions in nutrition (p = 0.01) and physical function (p < 0.001) domains. Geriatric assessment with geriatric intervention can be practically integrated into the routine care of older adults with HMs.

## Introduction

The median onset for hematologic malignancies is ~70 years of age (1). Advancing age is associated with inferior overall survival (OS) across all adult hematologic malignancies (HMs). This association between age and survival is complex and is impacted by comorbidities, geriatric syndromes, functional impairment, biological profiles, treatment decisions, and tolerance to therapy (2). Increasing use of a geriatric assessment (GA) to quantify and qualify the physiological age provides valuable information about treatment tolerability and OS across multiple cancer types (3, 4). A GA is a comprehensive multidomain evaluation utilized to identify occult vulnerabilities in older adults. Beyond prognostication, a GA is designed to direct therapeutic intervention. For older adults with solid tumors, the use of a GA and optimization has led to less treatment-related toxicity (5, 6), improved quality of life, and lower healthcare resource utilization (7) in randomized controlled trials. Integrating GA with geriatric interventions for patients with HM is an unmet need.

Frailty is characterized as a stepwise process of decline in reserves across multiple organ systems and can be precipitated by acute events, such as infection, falls, or any need for hospitalization (8). Frailty among older adults with myelodysplastic syndrome (MDS), acute myeloid leukemia (AML), lymphoma, or multiple myeloma (MM) has been associated with worse disease-related outcomes compared to more fit patients of similar chronological age (9). Frailty in older adults with HM may be related to the immunological or hematopoietic effects of cancer, toxicity related to treatment, and to accumulation of comorbid conditions prior to diagnosis. Fried’s frailty phenotype and Rockwood’s Clinical Frailty Scale (CFS) are among the most commonly used measures to characterize frailty (10, 11). Several HM-specific frailty screening tools are available; however, their widespread use and incorporation into routine care is limited. Brief in-person assessments such as gait speed and grip strength have been associated with worse survival among patients with HMs (12). While brief assessments are predictive of survival, a comprehensive multidisciplinary GA provides a domain-specific evaluation allowing for a focus on geriatric intervention.

We have incorporated GA with geriatric interventions into the care of older adults with HMs as part of routine cancer care delivery. Application of GA with geriatric-specific interventions was evaluated in a unique multidisciplinary oncogeriatric clinic model where older adults were seen and evaluated by a seven-member multidisciplinary team. Geriatric assessments were characterized, and interventions were prescribed with the objective of personalizing and optimizing patient health. Here, we report the real-world outcomes of older adults using a comprehensive GA in routine healthcare delivery, characterizing geriatric deficits with geriatric interventions.

## Methods

This real-world analysis includes all patients seen consecutively from February 1, 2016 to October 1, 2019 in the outpatient multidisciplinary oncogeriatric clinic, named Cancer and Aging Resiliency (CARE) clinic, at The Ohio State University Comprehensive Cancer Center/James Cancer Hospital. The CARE clinic consultative model is a novel approach integrating cancer subspecialties with oncogeriatric healthcare delivery and has previously been published (13). Patients were identified for evaluation by their treating hematologist and referred to the CARE clinic for a one-time consultation. Eastern Cooperative Oncology Group (ECOG) performance status, as documented by the referring physician, and laboratory data were collected when available within 30 days of CARE clinic consultation.

Study data were collected and managed using REDCap electronic data capture tool. Geriatric conditions were identified at the time of patient evaluation by the CARE physician and categorized into nine domains: functional impairment, falls/fall risk, cognitive impairment, poor psychosocial state, lack of social support or caregiver concerns, nutritional deficits, polypharmacy or inappropriate medication use, hearing or vision impairment, and financial stressor. Frailty was assessed by the CARE physician at the time of the patient evaluation according to the Rockwood CFS (14).

Objective measures of physical function included activities of daily living (ADL) (15), instrumental ADL (16), Functional Gait Assessment (FGA) (17), and/or the Timed Up and Go (TUG) test (18). Physical impairment was defined as FGA score <22 or TUG time ≥14 s. FGA was initially conducted for all patients, but removed from the CARE GA starting in July 2018 due to time constraints. ADL/IADL dependence was defined by lack of independence in any one or more domain on each respective scale. The Blessed Orientation Memory and Concentration (BOMC) test (19) or the Montreal Cognitive Assessment (MOCA) (20) was utilized to screen for cognitive impairment, defined as BOMC >4 (21) or MOCA <26 (20). BOMC was initially the preferred objective measure of cognitive function but was replaced by MOCA in January 2019. Self-report of exhaustion (yes/no) and physical health interference with social activities (e.g., all of time, some of the time, none) were assessed through patient interview as described in the Hurria GA (22).

Objective measure of nutrition status was the short-form Mini Nutritional Assessment (MNA). Objective measures of medication management included reporting of pharmacist-identified drug-therapy problems (23), including identification of potentially inappropriate medications by BEERS criteria (24). Additional details regarding audiology, nutrition, and medication management assessments have been previously described (13).

Descriptive statistics were used to summarize the clinical characteristics. For between-group comparisons, chi-squared test or Fisher exact test was used to compare categorical variables, and Kruskal–Wallis test was used to compare continuous variables. OS was measured from the date the patient first attended the CARE clinic to the date of death due to any cause, censoring patients who were still alive at the time of last follow-up. The method of Kaplan–Meier was used to describe the survival probability, which was compared between groups through the log-rank test. Cox proportional hazards models were built to assess the association between various clinical factors and OS, where univariable models for each variable were first fit; then, the backward selection method was used to construct the multivariable model with only statistically significant variables remaining in the final model. The proportional hazards assumption was checked to make sure that it was not violated for the model. Due to a relatively large amount of missing data for variables such as ECOG performance status, multiple imputation was used to build 50 imputed datasets, and the uni- and multivariable model results were obtained from combining the estimates and inferences across 50 imputed datasets. SAS software version 9.4 was used for statistical analysis. All tests were two-sided, and statistical significance was declared at α = 0.05.

## Results

### Patient Characteristics

The total study sample included 311 patients with diverse HM diagnoses including acute leukemia (AL, n = 38, 12%), myelodysplastic syndrome/myeloproliferative neoplasm/bone marrow failure (MDS/MPN/BMF, n = 47, 15%), plasma cell disorder (PCD, n = 108, 35%), lymphoma (n = 67, 22%), and chronic lymphocytic leukemia (CLL, n = 51, 16%). The study cohort was predominately white (n = 282, 91%) with a median age of 76 years (range, 57–95 years) and slightly more men than women (56% vs. 44%). At the time of evaluation, most patients lived with a significant other or spouse (n = 207, 67%), while a smaller proportion lived alone (n = 64, 21%). Most patients were actively being treated for their malignancy (n = 216, 77%) or had previously received treatment (n = 59, 21%). More than a quarter (n = 91, 29%) were under consideration for hematopoietic cell transplant or cellular therapy at the time of consultation. Additionally, one-quarter of patients (n = 82, 26%) had a second malignancy in the past or at the time of evaluation. The median hemoglobin in the cohort was 10.8 g/dl (range, 6.3–17 g/dl). The majority of patients referred to the CARE clinic were reported to have ECOG performance status of 0–1 (ECOG 0: n = 81, 34%; ECOG 1: n = 115, 49%) as identified by their primary hematologist within 30 days of GA.

### Geriatric Assessment

The summary of GA by HM diagnosis is presented in **Table 1** and includes geriatric deficits, Rockwood CFS, and objective measures of physical function, cognition, nutrition, and polypharmacy.

**Table 1:** Geriatric assessment and survival stratified by hematologic malignancy.

	Overall	Acute Leukemia	MDS/MPN/BMF	PCD	Lymphoma	CLL
N, no (%)	311	38 (12)	47 (15)	108 (35)	67 (22)	51 (16)
Age						
Median	76	69	71	78	75	80
Range	57-95	58-83	60-87	63-90	57-95	66-94
Sex, no (%)						
Female	138 (44)	11 (29)	11 (23)	59 (55)	35 (52)	22 (43)
Male	173 (56)	27 (71)	36 (77)	49 (45)	32 (48)	29 (57)
Treatment Status, no (%)						
On observation	7 (2)	0 (0)	1 (2)	3 (3)	1 (2)	2 (5)
On treatment	216 (77)	32 (89)	40 (95)	82 (83)	41 (63)	21 (53)
Pre. Treated	59 (21)	4 (11)	1 (2)	14 (14)	23 (35)	17 (43)
Unknown	29	2	5	9	2	11
ECOG Performance Status, no (%)						
0	81 (34)	19 (53)	16 (43)	18 (23)	15 (26)	13 (48)
1	115 (49)	15 (42)	20 (54)	41 (52)	27 (47)	12 (44)
2	32 (14)	2 (6)	1 (3)	18 (23)	10 (18)	1 (4)
3	8 (3)	0 (0)	0 (0)	2 (3)	5 (9)	1 (4)
Unknown	75	2	10	29	10	24
Rockwood CFS, no (%)						
Very fit/well	80 (26)	13 (35)	21 (45)	24 (22)	10 (15)	12 (24)
Managing well	106 (34)	13 (35)	19 (40)	32 (30)	26 (39)	16 (31)
Vulnerable	78 (25)	10 (27)	5 (11)	34 (32)	14 (21)	15 (29)
Frail	44 (14)	1 (3)	2 (4)	17 (16)	16 (24)	8 (16)
Unknown	3	1	0	1	1	0
^a^Cognitive Impairment	95	13	8	36	25	13
Unknown	25	3	5	9	5	3
^b^Physical Impairment	98	5	4	43	26	20
Unknown	136	25	26	41	31	13
MNA Score, no (%)						
Malnourished	34 (14)	4 (11)	3 (7)	12 (15)	13 (24)	2 (7)
At risk of malnutrition	106 (44)	23 (66)	18 (43)	29 (37)	27 (49)	9 (32)
Normal nutrition	99 (41)	8 (23)	21 (50)	38 (48)	15 (27)	17 (61)
Unknown	72	3	5	29	12	23
Number of current medications						
Median	11	12	9	12	12	11
Range	0-30	2-21	1-30	0-26	5-23	1-22
Unknown	37	4	4	21	8	0

MDS, myelodysplastic syndromes; MPN, myeloproliferative neoplasms; BMF, bone marrow failure; PCD, plasma cell myeloma; CLL, chronic lymphocytic leukemia; ECOG, Eastern Cooperative Oncology Group; CFS, clinical frailty scale; MNA, Mini Nutritional Assessment.

a Cognitive Impairment defined as The Blessed Orientation Memory and Concentration (BOMC) test >4 or the Montreal Cognitive Assessment (MOCA) <26.

b Physical Impairment defined by either Functional Gait Assessment (FGA) score <22 or Timed Up and Go Test (TUG) time ≥14 s.

#### General Fitness Assessment

**Figure 1:**
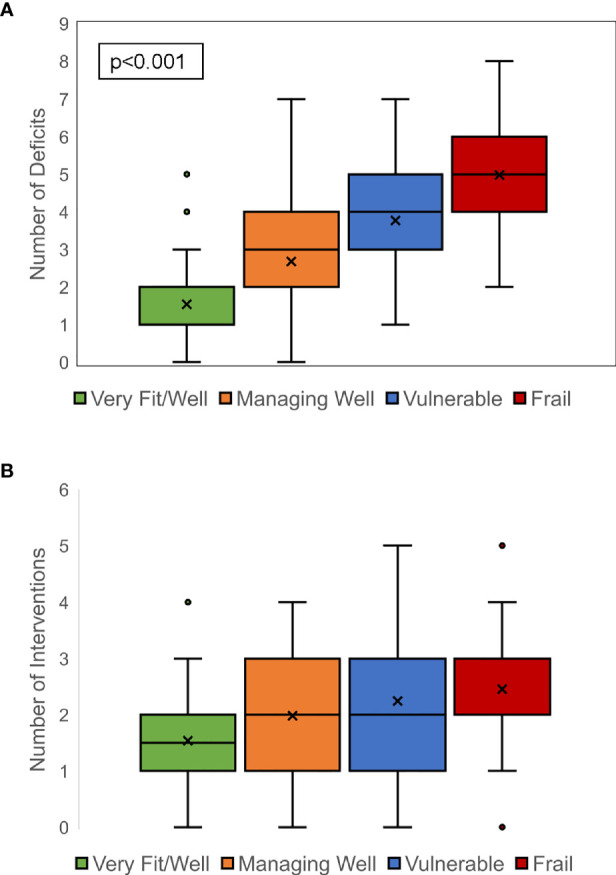
Correlation between clinical frailty scale rating and geriatric deficits and interventions. A strong correlation between increasing frailty and greater number of geriatric deficits was noted **(A)**. Similarly, a strong correlation between increasing frailty and greater number of prescribed geriatric interventions was seen **(B)**.

The median number of CARE physician-identified geriatric conditions was 3 (range, 0–8). Only 18 (6%) patients had 0 identified geriatric conditions. Frailty was characterized by the CFS, categorized patients into four groups: very fit/well (n = 80, 26%), managing well (n = 106, 34%), vulnerable (n = 78, 25%), and frail (n = 44, 14%). Patients with PCD had the highest percentage of CFS vulnerable rating (n = 34, 32%), followed by CLL (n = 15, 29%) and AL (n = 10, 27%). Patients with lymphoma had the highest percentage of CFS mildly, moderately, or severely frail rating (n = 16, 24%), followed by PCD (n = 17, 16%) and CLL (n = 8, 16%). Geriatric conditions were common, and the number of geriatric conditions was highly correlated with CFS frailty categories (**Figure 1A**). The median number of geriatric deficits was 1, 3, 4, and 5, respectively, in patients identified by CFS as being very fit/well, managing well, vulnerable, and frail (p < 0.001).

#### Physical Function

Thirty-four patients (11%) reported ADL dependence with the most common impairments in transferring (n = 25/34, 74%) and grooming (n = 20/34, 59%). IADL dependence was present in 123 patients (41%). The most common IADL impairments included using transportation (n = 94/123, 76%), doing housework (n = 89/123, 72%), and shopping (n = 84/123, 68%). Physical impairment, as measured by either FGA score or TUG time, was found in 98 (56%) patients where the data were available. TUG test showed significant variability with a median of 10 s and a range of 5–79 s. Additionally, 171 patients (56%) were already using an assistive device at the time of assessment.

#### Cognitive, Sensory, and Psychosocial Assessment

One-third of patients (n = 95, 33%) screened positive for cognitive impairment by either BOMC or MOCA. Hearing loss was prevalent with 216 (71%) patients with hearing deficit identified, yet only 58 (27%) of them were already wearing hearing aids. Firearm use was found in 38 of 169 evaluable patients (22%). Exhaustion was reported by 84 (28%) patients, and physical health interference with social activities was reported some, most, or all of the time in 137 (47%) patients. Thirty-seven (12%) patients had psychosocial concerns identified by nurse case manager.

#### Nutrition and Medication Management

Nutritional impairments, as measured by MNA, were identified in more than half of patients with 34 (14%) suffering from malnourishment and 106 (44%) patients at-risk for malnourishment. A third of the patients (n = 104, 35%) self-reported weight loss. Median body mass index was 26.8 kg/m^2^ (range, 15–52.8). Medication reconciliation revealed 480 pharmacist-identified and 154 patient-identified drug-therapy problems for a total of 634 drug-therapy problems among 268 (86%) patients with any drug therapy concerns. Forty-six patients (15%) had no pharmacist-identified drug-therapy problems. The median number of current medications not including chemotherapy was 11 (range, 0–30). The use of anticoagulants was found in 179 patients (60%). The most common categories of BEERS criteria inappropriate medications were gastrointestinal including proton-pump inhibitors and metoclopramide (n = 86, 28%), benzodiazepines (n = 33, 11%), and anticholinergics (n = 25, 8%).

### Overall Survival

**Figure 2:**
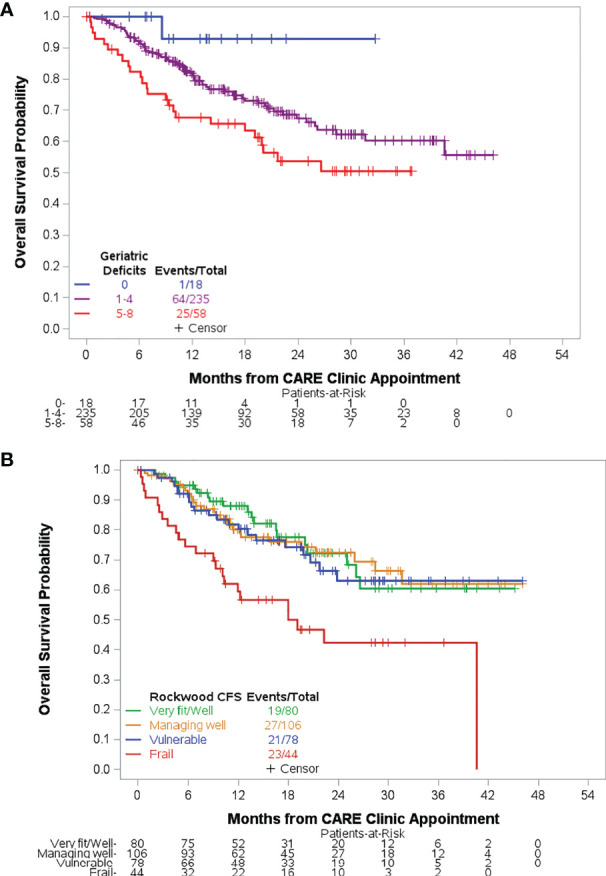
Overall survival based on measures of overall health. Accumulation of geriatric deficits was associated with inferior overall survival **(A)**. Patients with mild, moderate, or severe frailty by CFS experienced inferior overall survival compared to fit patients. There was no significant difference in survival for managing well or vulnerable compared to fit patients **(B)**.

With a median follow-up time of 17.7 months (range, 0–46.1) among 221 survivors, the median OS from time of CARE clinic evaluation has not been reached. Median OS for each HM diagnosis type is found in **Table 1**. In univariable analysis, disease type (AL, MDS/MPN/BMF), older age, accumulation of geriatric conditions, CFS frailty, increasing TUG score, ADL dependence, decreasing hemoglobin, and less-than-normal nutrition status were associated with worse OS (**Table 2**). OS was worst among patients with five or more geriatric conditions (**Figure 2A**) and among patients rated mildly, moderately, or severely frail by CFS (**Figure 2B**). In multivariable analysis, HM diagnosis (AL, HR = 3.84, 95% CI 1.60–9.22, p = 0.003; MDS/MPN/BMF, HR = 2.65, 95% CI 1.10–6.35, p = 0.03), age (per 5-year increase, HR = 1.46, 95% CI 1.21–1.76, p < 0.001), hemoglobin (per 1 g/dl decrease, HR = 1.21, 95% CI 1.05–1.40, p = 0.009), ADL dependence (HR = 2.20, 95% CI 1.11–4.34, p = 0.02), and MNA score (at-risk of malnutrition vs. normal, HR = 2.00, 95% CI 1.07–3.73, p = 0.03) were independently associated with risk of death.

**Table 2:** Univariable and Multivariable analysis of overall survival.

	Univariable		Multivariable	
	Hazard Ratio (95% CI)	p-value	Hazard Ratio (95% CI)	p-value
Disease vs. CLL				
Acute leukemia	2.99 (1.40-6.38)	0.005	3.84 (1.60-9.22)	0.003
Lymphoma	1.20 (0.58-2.47)	0.63	0.69 (0.30-1.57)	0.38
MDS/MPN/BMF	2.39 (1.16-4.92)	0.02	2.65 (1.10-6.35)	0.03
PCD	1.22 (0.63-2.38)	0.55	1.02 (0.50-2.07)	0.95
Age, 5-year increase	1.17 (1.01-1.36)	0.03	1.46 (1.21-1.76)	<.0001
ECOG PS, 1-unit increase	1.17 (0.88-1.55)	0.28	—	—
Treatment status, vs. never treated, on observation				
On treatment	0.77 (0.18-3.25)	0.72		
Previously treated	0.90 (0.20-3.99)	0.89		
Self-reported exhaustion	1.02 (0.65-1.61)	0.94	—	—
Total number of deficits, 1-unit increase	1.13 (1.00-1.27)	0.04	—	—
Frailty assessment summary, vs. very fit/well				
Managing well	1.07 (0.59-1.93)	0.82		
Vulnerable	1.15 (0.62-2.15)	0.66		
Frail (mildly/moderately/severely)	2.59 (1.41-4.78)	0.002		
ADL dependence	2.11 (1.22-3.63)	0.008	2.20 (1.11-4.34)	0.02
IADL dependence	1.47 (0.96-2.24)	0.07	—	—
Hemoglobin, 1-unit decrease	1.26 (1.31-1.42)	<.0001	1.21 (1.05-1.40)	0.009
BMI, 5-unit increase	0.83 (0.68-1.01)	0.07	—	—
Self-reported weight Loss	1.38 (0.88-2.15)	0.16		
Number of pharmacist-identified drug therapy problems	1.00 (0.85-1.19)	0.98	—	—
Number of current medications (excluding chemotherapy)	1.02 (0.98-1.07)	0.24	—	—
Time up and go, 3-second increase	1.08 (1.01-1.15)	0.02	—	—
Physical Impairment (by TUG or FGA)	1.63 (0.98-2.69)	0.06	—	—
MNA, vs normal nutrition				
malnourished	2.72 (1.39-5.34)	0.004	2.01 (0.94-4.28)	0.07
at risk of malnutrition	2.21 (1.24-3.96)	0.008	2.00 (1.07-3.73)	0.03
Cognitive Impairment (by BOMC or MOCA)	1.31 (0.83-2.06)	0.24	—	—

CI, confidence interval; CLL, chronic lymphocytic leukemia; MDS, myelodysplastic syndromes; MPN, myeloproliferative neoplasms; BMF, bone marrow failure; PCD, plasma cell myeloma; ECOG PS, Eastern Cooperative Oncology Group performance status; ADL, activities of daily living; IADL, instrumental activities of daily living; BMI, body mass index; TUG, Time Up And Go; FGA, Functional Gait Assessment; MNA, Mini Nutritional Assessment; BOMC, Blessed Orientation–Memory–Concentration; MOCA, Montreal Cognitive Assessment.

### Geriatric Interventions

**Figure 3:**
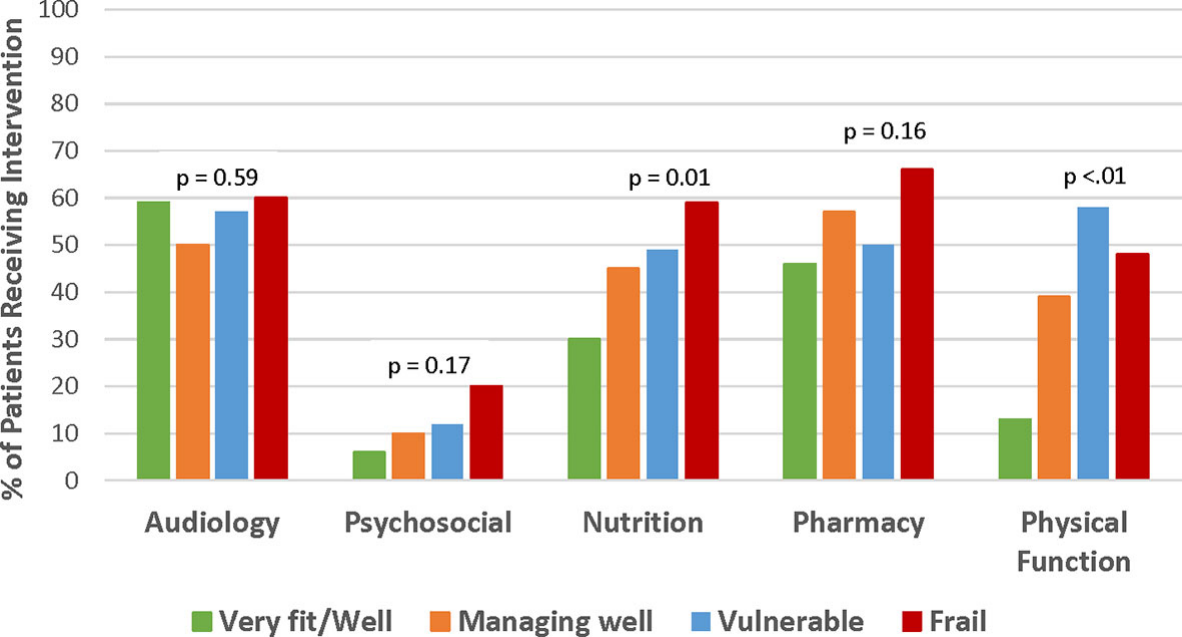
Frequency of prescribed interventions by CFS frailty rating. Managing well and vulnerable patients was the more frequently prescribed intervention across all domains except for audiology where intervention frequency was similar across all groups. Interventions in psychosocial domain were less frequently prescribed compared to other domains.

This analysis focused on the identification of commonly occurring geriatric conditions and specific interventions prescribed or recommended in the CARE clinic. The number of geriatric interventions was significantly correlated with frailty status (p < 0.001, **Figure 1B**). The median number of interventions increased from 1.5 to 2, 2, and 3 in patients who were very fit/well, managing well, vulnerable, and frail, respectively. Interventions were clustered by domain. No interventions were recommended for 22 (7%) patients. In general, across all domains except audiology, the proportion of patients receiving intervention increased with a more severe degree of frailty, especially in nutrition (p = 0.01) and physical function (p < 0.01) (**Figure 3**). Audiology and pharmacy interventions were recommended in similar proportions of patients across CFS groups.

Audiology was the domain most commonly intervened upon (n = 171, 56%). Otology referral was indicated for 106 (35%) patients. Of the 158 hearing impaired patients without hearing aids, 137 (87%) were recommended to pursue hearing aid evaluation. Pharmacy interventions, including de-prescribing, dose adjustment, new prescription, and suggested medication modification, were indicated in 168 (54%) patients. Nutrition supplementation was prescribed for 136 (44%) patients. Referrals for rehabilitative therapy were made for 110 (37%) patients with the majority going to physical therapy (n = 105, 95%) and an additional 5 to occupational therapy, 1 to speech therapy, and 4 to aquatic therapy. Ten (7%) patients were prescribed an assistive device among 135 patients who were not using one at the time of evaluation. Of the 95 patients with cognitive impairment, 4 (4%) were referred to neuropsychiatric services. Among 37 patients with case management needs identified, 16 (44%) were referred for financial counseling, 9 (24%) for home-community-based resources for older adults, and 12 (32%) for ongoing follow-up with social worker in primary hematology clinic.

## Discussion

In this real-world analysis of multidomain, consultative GA in older adults with HMs, the accumulation of geriatric conditions correlated with the Rockwood CFS rating. Frailty and increasing number of geriatric conditions were predictive of worse OS. Despite heterogeneity in the type of geriatric conditions, these cumulative measures of overall health capture a subset of older adults with HMs at highest risk of mortality. We have shown the individual domains of physical fitness and nutrition, as measured by ADL dependence and MNA scores, to correlate with OS.

In our sample, we found differences in the presence of geriatric conditions based on underlying HM. In particular, patients with AL or MDS/MPN/BMF were younger, more likely to be male, less frail by the CFS, and with less physical impairment than the other disease groups, although their median OS was shorter. Patients with lymphoma or CLL were less likely to have received previous treatment than the other disease groups, and their median OS was longer. GA results in patients with PCD were most heterogeneous. Characteristics inherent to the diseases themselves (typical course and symptomatology) and to their treatments (targeted therapy vs. cytotoxic chemotherapy) likely play a role in these differences. Importantly, some patients were referred for oncogeriatric services based on perceived need, while others were referred as part of a standardized evaluation (e.g., prior to hematopoietic cell transplant or cellular therapy). Identification of these emerging patterns of geriatric conditions based on the underlying HM can help guide care in subspecialty disease clinics. For example, high rates of IADL dependence and physical impairment in patients with PCD or lymphoma may warrant development of a home-based exercise program (25). A recent work from Loh et al. provides qualitative insight into preferences of patients with myeloid neoplasms for developing a mobile health-based program (26). Future prospective cohort studies underway in HM will provide further insight into the ideal use of a GA partnered with geriatric interventions.

In a recent systematic review of GA in patients with HM, deficits in ADL and IADL were commonly associated with OS, with 67% and 74% reporting a significant association in univariate and 40% and 62% in multivariate analysis, respectively (4). In the same review, the prevalence of IADL dependence (45%) was similar to our findings (41%), although we found only a borderline association with OS (p = 0.07). One possibility for this difference is that our clinic is prescriptive with respect to interventions. As an example, a patient with IADL dependence related to physical impairment will be referred to physical therapy at the time of GA evaluation with same-day scheduling. It is possible that we are measuring the effect, not just of an impairment or geriatric condition present at GA but also of the prescribed intervention. One large-scale study of older Belgian patients with cancer demonstrated an excellent adherence rate to prescribed interventions (64%), measured at a 3-month follow-up visit; however, the authors acknowledged the great difficulty and expense that would be associated with long-term follow-up to determine the effect of adherence to prescribed intervention (27). Of 44 relevant studies of GA in patients with HM identified in the previously cited systematic review, 36 reported ADL, IADL, or both scores. These measures have been well characterized as predictors of survival, and we would continue to recommend this assessment in oncogeriatric clinics (4).

The majority of patients were identified to have a favorable performance status, yet the prevalence of geriatric conditions was high. We have shown high prevalence of nutritional (59%), physical (56%), sensory (71%), and cognitive (33%) impairments, and pharmacist-identified drug-therapy problems (85%). Physician-rated performance status has previously been shown to predict OS in patients with HMs (28, 29), although it was not a significant predictor in this cohort of older adults with HM. Not all identified geriatric conditions lead to clinically significant frailty and may not be accounted for by ECOG performance status rating. Importantly, a GA is recommended for all adults 65 years or older by standing guidelines (30–32) and major societies (33, 34), yet the routine uptake of GA in routine oncology care has been stunted. GA healthcare delivery implementation barriers include system constraints such as limited resources, provider barriers including paucity of geriatricians, perceived time constraints in resources, knowledge gaps, timing of GA delivery, and patient barriers including travel.

This CARE clinic model was built around a multidisciplinary team led by hematologists/oncologists with expertise in needed disciplines (nutrition, physical therapy, audiology, case management, pharmacy) and colocated physically within routine hematology care delivery. This alignment in physical space, consultative model, and ownership of clinical interventions allowed for a robust referral network. The GA metrics outlined within this model are one example, and many other GA tools and models are well established to identify vulnerabilities in specific patient populations. The Cancer and Aging Research Group (CARG) has developed an electronic data capture GA tool that is largely comprised of patient-reported data including ADLs, IADLs, falls, limitations in social activity, comorbid conditions, and number of current medications; psychological state assessed by Mental Health Inventory; and supplemented by physician-rated performance status, TUG, and BOMC scores. Utilizing data from this GA, CARG has also developed a calculator tool predictive of chemotherapy toxicity (22). Another similar tool to predict chemotherapy toxicity, Chemotherapy Risk Assessment Scale for High-Age Patients (CRASH), was developed by Extermann et al. (35). Neither the CARG nor CRASH tools have been validated in the HM population specifically.

One limitation of this study was that, while the assessed domains remained the same for each patient, measurement tools changed over time. As an example, the BOMC was initially used for simplicity; however, new data suggested that MOCA was more effective for identifying cognitive changes and replaced BOMC in our assessment (36). Several frailty scales are available including Frieds frailty index and others that are disease specific like the Myeloma Frailty Score (37), validated in patients with multiple myeloma, or the Elderly Prognostic Index (38), validated in patients with diffuse large B-cell lymphoma. We recognize that a comprehensive GA is not a one-size-fits-all tool and have conveyed in our study the importance of assessing multiple domains of function and overall fitness, irrespective of which domain-specific tools are used. A second limitation of this study is that not all interventions were evaluated for adherence. As an example, de-prescription in the case of inappropriate medication use is a one-time intervention occurring within the GA visit, and medication list is updated in real-time to reflect this; however, interventions requiring referral, such as physical therapy, were documented as being ordered, but patient adherence could not be assessed. Lastly, many of the independent predictors of OS in our multivariate model include inherent patient and disease characteristics like chronological age, disease type, and hemoglobin, while the effect of potentially modifiable factors, like malnutrition and ADL dependence, is confounded by intervention.

To date, this is one of the largest studies of older adults with HM, describing the implementation of an oncogeriatric assessment partnered with interventions as part of routine oncology care.

To meet the needs of aging adults with HM, robust metrics are required, outside of traditional performance status, to proactively intervene for sustainable health with cancer treatment. In the future, a prospective cohort study of serial GA will yield a clearer picture of the modifiability of geriatric deficits and the effect of intervention on survival.

## Data Availability Statement

The original contributions presented in the study are included in the article/supplementary material. Further inquiries can be directed to the corresponding author.

## Ethics Statement

The studies involving human participants were reviewed and approved by Ohio University Cancer Institutional Review Board. The patients/participants provided their written informed consent to participate in this program study.

## Author Contributions

SW and AR contributed to concept and study design. SW, AF, CK, NY, EF, CP, and AR contributed to data acquisition. SW, YH, AK, and AR contributed to data analysis and interpretation. SW, YH, AF, TG, EF, ES, CP, NW, JK-S, MN, and AR contributed to manuscript preparation. All authors contributed to the article and approved the submitted version.

## Funding

Research reported in this publication was supported by the National Cancer Institute 1 K23 CA208010-01 (PI Rosko). The content is solely the responsibility of the authors and does not necessarily represent the official views of the National Institutes of Health.

## Conflict of Interest

MN receives funding from the Merck Foundation. TG has received funding from the National Institute on Aging, and National Institute of Heart, Lung, & Blood.

The remaining authors declare that the research was conducted in the absence of any commercial or financial relationships that could be construed as a potential conflict of interest.

## Publisher’s Note

All claims expressed in this article are solely those of the authors and do not necessarily represent those of their affiliated organizations, or those of the publisher, the editors and the reviewers. Any product that may be evaluated in this article, or claim that may be made by its manufacturer, is not guaranteed or endorsed by the publisher.
